# Functional characterization of the phosphotransferase system in *Parageobacillus thermoglucosidasius*

**DOI:** 10.1038/s41598-023-33918-1

**Published:** 2023-05-02

**Authors:** Gonzalo N. Bidart, Hani Gharabli, Ditte Hededam Welner

**Affiliations:** The Novo Nordisk Center for Biosustainability, Technical University of Denmark, Kemitorvet 220, DK-2800 Kgs. Lyngby, Denmark

**Keywords:** Industrial microbiology, Applied microbiology, Environmental microbiology

## Abstract

*Parageobacillus thermoglucosidasius* is a thermophilic bacterium characterized by rapid growth, low nutrient requirements, and amenability to genetic manipulation. These characteristics along with its ability to ferment a broad range of carbohydrates make *P. thermoglucosidasius* a potential workhorse in whole-cell biocatalysis. The phosphoenolpyruvate:carbohydrate phosphotransferase system (PTS) catalyzes the transport and phosphorylation of carbohydrates and sugar derivatives in bacteria, making it important for their physiological characterization. In this study, the role of PTS elements on the catabolism of PTS and non-PTS substrates was investigated for *P. thermoglucosidasius* DSM 2542. Knockout of the common enzyme I, part of all PTSs, showed that arbutin, cellobiose, fructose, glucose, glycerol, mannitol, mannose, *N*-acetylglucosamine, *N*-acetylmuramic acid, sorbitol, salicin, sucrose, and trehalose were PTS-dependent on translocation and coupled to phosphorylation. The role of each putative PTS was investigated and six PTS-deletion variants could not grow on arbutin, mannitol, *N*-acetylglucosamine, sorbitol, and trehalose as the main carbon source, or showed diminished growth on *N*-acetylmuramic acid. We concluded that PTS is a pivotal factor in the sugar metabolism of *P. thermoglucosidasius* and established six PTS variants important for the translocation of specific carbohydrates. This study lays the groundwork for engineering efforts with *P. thermoglucosidasius* towards efficient utilization of diverse carbon substrates for whole-cell biocatalysis.

## Introduction

In the past decades, industrial biotechnology and biocatalysis have become an increasingly integrated part of the synthesis and manufacturing of therapeutics and food additives, providing a selective and sustainable solution for modern production^1^. However, this solution is constantly under economic pressure caused by the high demand and competing performance of heterogeneous catalysis in the chemical industry^2^. A challenge of biocatalysis is the use of bacterial strains grown under mild conditions allowing the growth of unwanted microorganisms^3^. Here, extremophilic microorganisms have gained increasing interest due to their resilience towards extreme conditions—leading to the decreased risk of contamination—along with their ability to produce proteins and enzymes that retain function under extreme conditions^4^. Enzymes from thermophilic microorganisms, or thermozymes, have previously been highlighted for not only their thermostability but also their resistance towards chemical denaturing agents, wide pH tolerance, and non-aqueous solvents making them relevant for industrial applications^3^. The use of higher temperatures in industrial processes would also increase substrate/product solubility, reduce hydrolysis time, reduce cooling costs, and decrease the viscosity of the liquids used in the process^5^. Thermozymes have been utilized for *in vitro* single-step reactions, but more complex multistep chemical conversions are primarily done in an intact cellular host, and this approach has been halted by the lack of genetic tools for thermophilic organisms.

**Figure 1:**
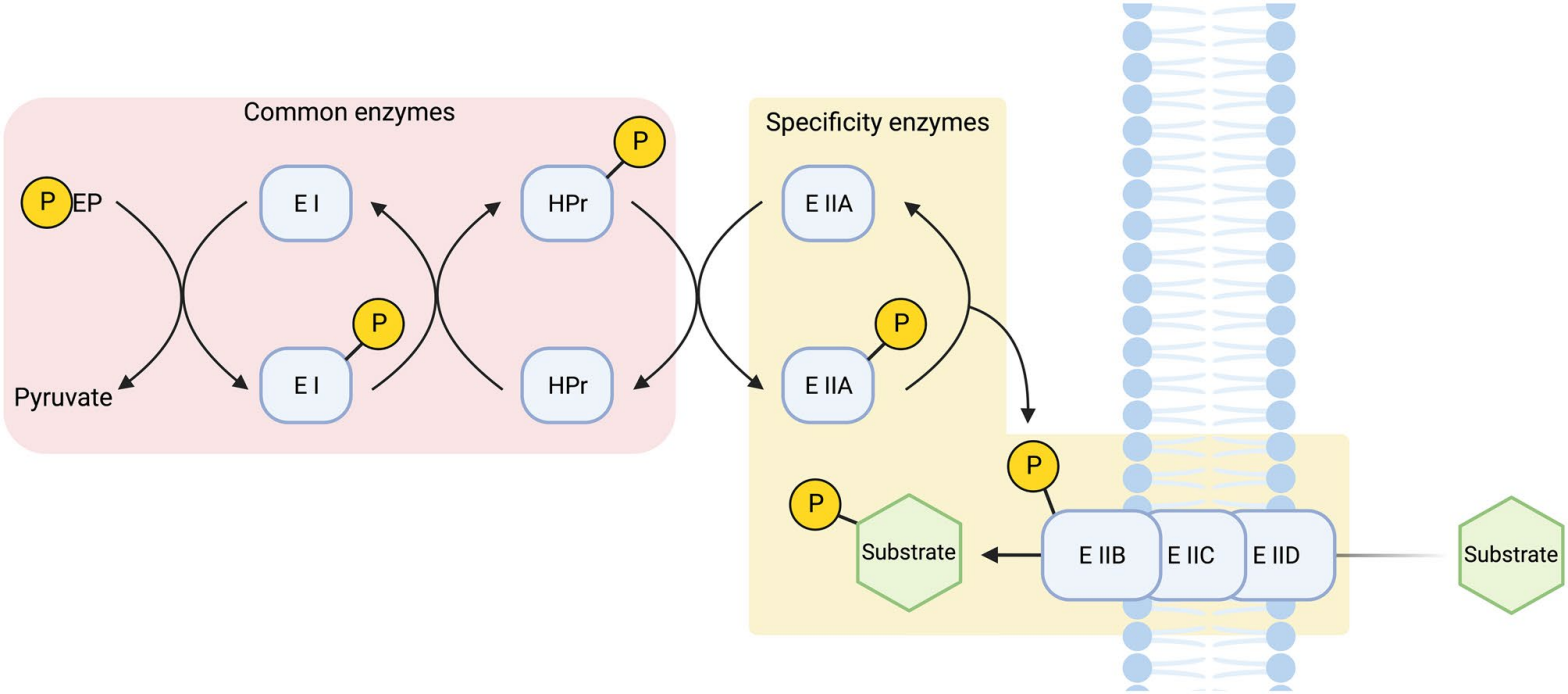
Schematic representation of the general bacterial phosphotransferase system (PTS). The two general cytoplasmatic components, which are used for all putative PTS systems are indicated in pink. These are the PTS-general component Enzyme I (EI) and the histidine-containing phosphocarrier protein (HPr). The area marked in tan is a representation of an Enzyme II (EII) complex. EII complexes confer the carbohydrate specificity and are specific for each PTS system. Each EII complex is formed either by distinct proteins or by a single multidomain protein and consists of two hydrophilic domains (EIIA and EIIB), and one or two transmembrane domains (EIIC and EIID).

*Parageobacillus* spp. are thermophilic aerobic or facultatively anaerobic Gram-positive bacilli capable of growth between 40 and 70 °C with an optimum temperature of 60–65 °C^6–8^. Members of the genus can ferment both hexose and pentose monosaccharides and oligosaccharides to generate lactate, formate, acetate, and ethanol as products^6^. As type species of the genus (*Parageobacillus*), *P. thermoglucosidasius* DSM 2542^9^ (*P. thermoglucosidasius* henceforth), has been engineered and utilized for industrial bioethanol production from lignocellulosic feedstocks^6^. Different works have also engineered this bacterium for the production of isobutanol^10^, riboflavin^11^, (S)-lactic acid^12^, terpenes^13^ and (2R, 3R)-butanediol^14^. The strain demonstrates a rapid growth rate and the ability to ferment a broad range of monosaccharides, cellobiose, and short-chain oligosaccharides. Its success in bioethanol production, the possibility of genetic manipulation, and recent whole-genome sequencing have highlighted *P. thermoglucosidasius* as a potential future cell factory for other valuable small molecules. To further increase the understanding and leveraging of *P. thermoglucosidasius,* it is necessary to characterize the metabolism of the microorganism, including its carbohydrate metabolism and transportation. In prokaryotes, the transport of carbohydrates is mainly catalyzed by the phosphoenolpyruvate(PEP):carbohydrate phosphotransferase system (PTS)^15^. The PTS couples the transport of carbohydrates with subsequent phosphorylation through a four-step phosphoryl transfer system^15,16^. Each PTS consist of two cytoplasmatic proteins, the PTS-general component Enzyme I (EI) that receives the phosphate from PEP, and the histidine-containing phosphocarrier protein (HPr), which is phosphorylated by EI along with a substrate-specific Enzyme II (EII) complex (Fig. 1)^15,16^. Generally, EI and HPr are common to all PTSs of a cell, meaning that they perform the phosphoryl transfer to all the different EII complexes. Each EII complex is formed by two cytoplasmatic domains; EIIA, which is phosphorylated by HPr, and EIIB, which is phosphorylated by EIIA; and one or two integral membrane domains (EIIC/EIID) that are necessary for substrate translocation (Fig. 1). Furthermore, the three or four EII domains could be either encoded in a single multi-domain protein, or in distinct single-domain proteins^15^.

The PTS participates in complex regulatory mechanisms, including both carbon and nitrogen metabolisms. In summary, in low G+C DNA Gram-positives, HPr also works as a sensor of glycolytic intermediates, especially for fructose-1,6-bisphosphate (FBP). High concentrations of FBP increase phosphorylation of HPr on the conserved serine-46 (different to the histidine involved in the PTS phosphorelay) triggering carbon catabolite repression (CCR), generally through the carbon catabolite repressor CcpA^15^. Furthermore, CcpA also regulates the synthesis of branched-chain amino acids which directly stimulates the global regulator CodY, linked to both carbon and nitrogen metabolism^17^. For detailed explanations of the regulatory networks refer to Deutscher *et. al* (2006) and Sonenshein (2007).

In this study, we investigated the role of the PTSs in the transport of common carbon substrates in *P. thermoglucosidasius*. By constructing PTS knockouts and measuring the growth of *P. thermoglucosidasius* on fifteen common carbon substrates, we were able to determine some of the PTSs specificities in *P. thermoglucosidasius* and show that there is a complex redundancy between different PTS systems. Of the fifteen substrates, thirteen showed a dependence on active PTS-mediated transport. Knockouts of a minimum of one PTS element in each of the fifteen putative PTS gene clusters in *P. thermoglucosidasius* revealed five PTSs solely responsible for the translocation of arbutin, mannitol, *N*-acetylglucosamine, sorbitol, and trehalose, respectively, and the main PTS responsible for the translocation of *N*-acetylmuramic acid. This study establishes the basis for further metabolic- and strain engineering of *P. thermoglucosidasius* for novel biotechnological solutions.

## Results

### Genome analysis and variant design

**Figure 2:**
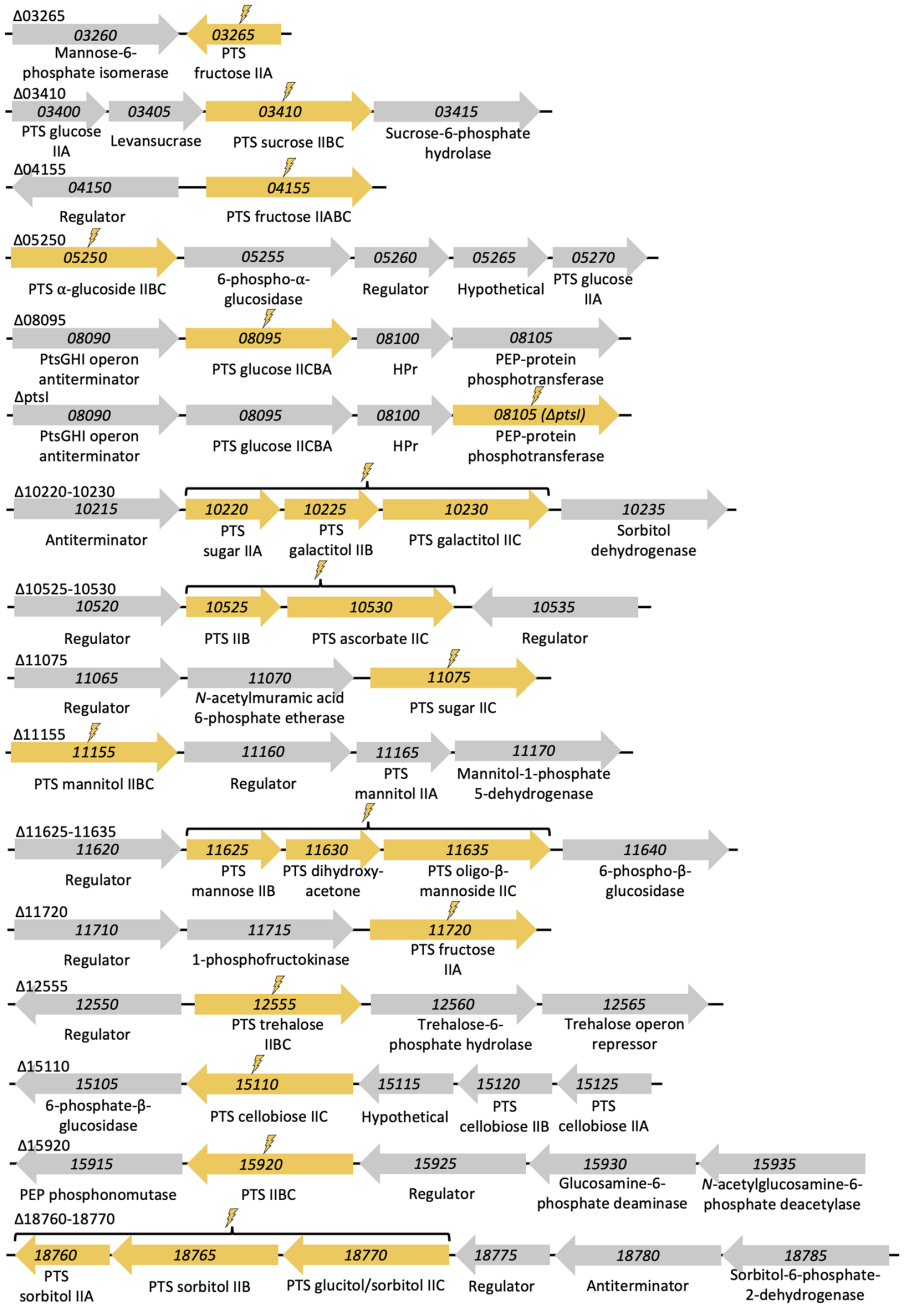
Gene clusters with at least one putative gene coding for a PTS element. Yellow thunderbolts indicate the gene(s) that were knocked out. The numbers correspond to the locus tag of each gene and below, the putative function of the protein encoded by the gene.

To assess the capacity of *P. thermoglucosidasius* DSM 2542 to metabolize carbon substrates through PTS and associated elements thereof, we searched on the available genome sequence (GenBank Accession No. CP012712)^9^ for genes encoding putative PTSs. The genomic analysis, performed as indicated in *Bioinformatic analysis* in the Materials and Methods section, revealed that *P. thermoglucosidasius* contains 15 genomic regions with at least one such PTS element (Fig. 2), scattered throughout the bacterial genome. A minimum of one PTS-associated gene from each gene cluster was knocked out by a scarless-genome edition method based on allelic replacement^6^, yielding 15 deletion strains. In addition, the gene encoding the EI (*ptsI*; AOT13_08105) (Fig. 1) was also knocked out, thereby yielding a total of 16 deletion variants of *P. thermoglucosidasius* (Fig. 2 and Supplementary Fig. 16).

### Knockout of *ptsI*

**Figure 3:**
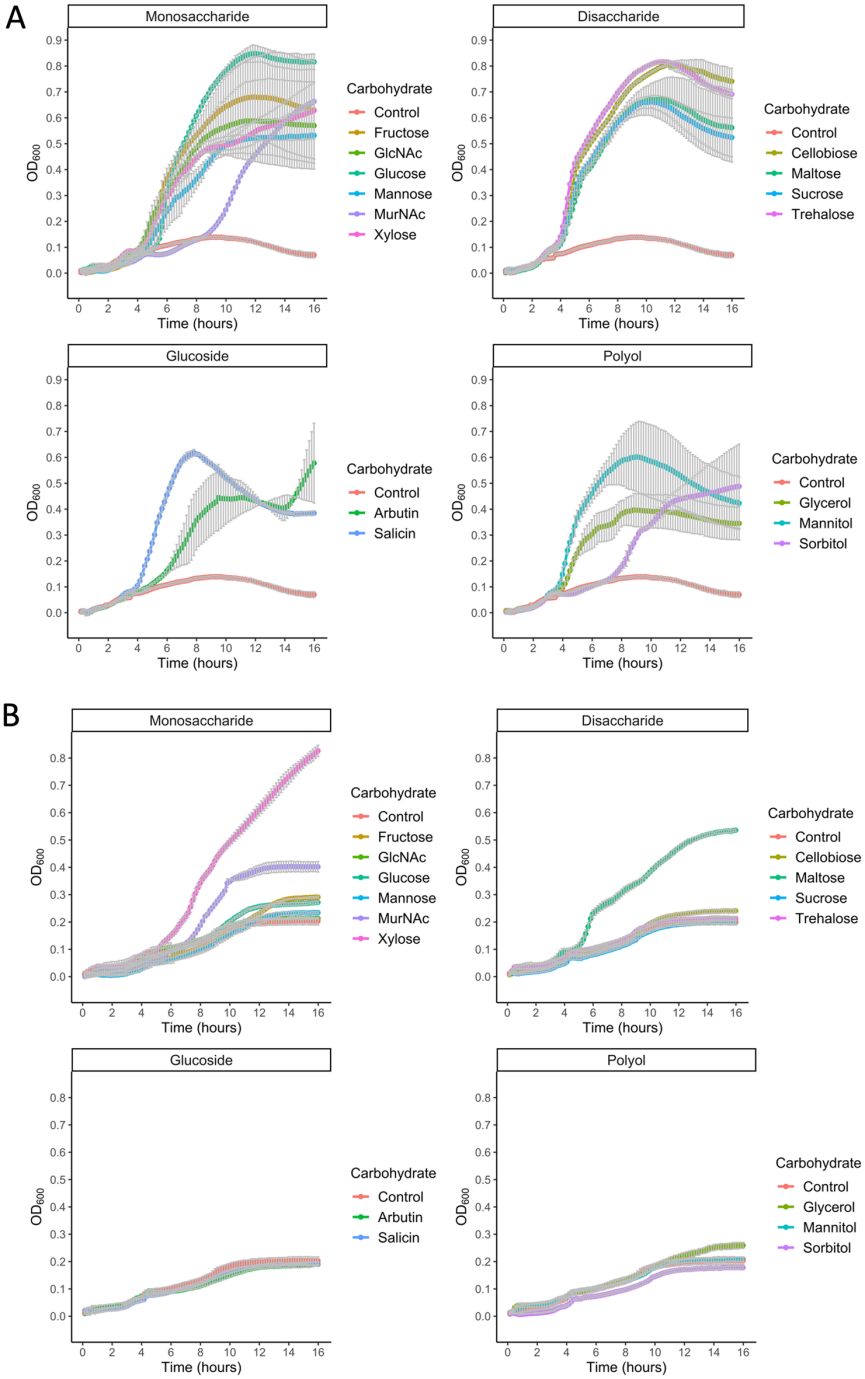
Growth curves of *P. thermoglucosidasius* DSM 2542 wild type strain (**A**) and ∆*ptsI* (**B**) on TMMYE medium without (control) or with supplemented carbohydrates (Fructose, Glc*N*Ac, Glucose, Mannose, Mur*N*Ac, Xylose; Cellobiose, Maltose, Sucrose, Trehalose; Arbutin, Salicin; Glycerol, Mannitol or Sorbitol). Data presented are average values based on at least three replicates. Error bars indicate standard deviations.

After the mutants were constructed, we focused on quantitative physiology experiments towards identifying the associated growth phenotypes. To this end, 15 carbon sources were selected for their potential as substrates for *P. thermoglucosidasius*. Besides the carbon sources typically used for bacterial growth experiments (e.g., glucose, fructose, mannose, and xylose), other substrates were likewise included (*N*-acetylglucosamine, *N*-acetylmuramic acid, glycerol, mannitol, sorbitol, cellobiose, maltose, sucrose, trehalose, arbutin, and salicin). To determine which of the 15 substrates were transported by the PTS in *P. thermoglucosidasius*, the WT strain along with the common enzyme knockout strain, ∆*ptsI* (strain GTS17 in Table 1), were grown in minimal media supplemented with the 15 carbon substrates, respectively. Comparing the growth profiles of these two strains revealed that the WT strain grew in all conditions (Fig. 3A), while the ∆*ptsI* deletion mutant had abolished growth with 12 out of the 15 feedstocks (Fig. 3B). The ∆*ptsI* strain had no significant growth when the medium was supplemented with arbutin, cellobiose, fructose, glucose, glycerol, mannitol, mannose, *N*-acetylglucosamine, sorbitol, salicin, sucrose, or trehalose. These results indicate PTS-dependent processing of the 12 molecules. Although we cannot exclude that other functional elements could be involved in the transport of the substrates tested herein (e.g., hexose permeases or facilitators, which could promote growth on glucose or fructose)^18–20^. In addition to the 12 carbon substrates on which growth was abolished, *N*-acetylmuramic acid yielded diminished growth indicating that this substrate is mainly, but not exclusively, metabolized through PTS. Interestingly, the ∆*ptsI* strain was still able to grow when the media was supplemented with maltose or xylose. These results indicate that the transport of these sugars is either independent of phosphorylation processes catalyzed by PTSs or, as indicated above, could be mediated by another transport mechanism, such as a permease.

**Table 1:** Strains used in this study.

Strain	Relevant genotype or properties	Source
DSM 2542	*P. thermoglucosidasius* Wild type	Suzuki *et al.* 1983^53^ Aliyu *et al.* 2016^54^; Bacillus Genetic Stock Center (USA)
GTS1	DSM 2542 *ptsI*::pGeo Kan^R^	This work
GTS2	DSM 2542 *03265*::pGeo Kan^R^	This work
GTS3	DSM 2542 *03410*::pGeo Kan^R^	This work
GTS4	DSM 2542 *04155*::pGeo Kan^R^	This work
GTS5	DSM 2542 *05250*::pGeo Kan^R^	This work
GTS6	DSM 2542 *08095*::pGEO Kan^R^	This work
GTS7	DSM 2542 *10220–10230*::pGeo Kan^R^	This work
GTS8	DSM 2542 *10525–10530*::pGeo Kan^R^	This work
GTS9	DSM 2542 *11075*::pGeo Kan^R^	This work
GTS10	DSM 2542 *11155*::pGeo Kan^R^	This work
GTS11	DSM 2542 *11625–11635*::pGeo Kan^R^	This work
GTS12	DSM 2542 *11720*::pGeo Kan^R^	This work
GTS13	DSM 2542 *12555*::pGeo Kan^R^	This work
GTS14	DSM 2542 *15110*::pGeo Kan^R^	This work
GTS15	DSM 2542 *15920*::pGeo Kan^R^	This work
GTS16	DSM 2542 *18760–18770*::pGeo Kan^R^	This work
GTS17	DSM 2542 Δ*ptsI*	This work
GTS18	DSM 2542 Δ*03265*	This work
GTS19	DSM 2542 Δ*03410*	This work
GTS20	DSM 2542 Δ*04155*	This work
GTS21	DSM 2542 Δ*05250*	This work
GTS22	DSM 2542 Δ*08095*	This work
GTS23	DSM 2542 Δ*10220–10230*	This work
GTS24	DSM 2542 Δ*10525–10530*	This work
GTS25	DSM 2542 Δ*11075*	This work
GTS26	DSM 2542 Δ*11155*	This work
GTS27	DSM 2542 Δ*11625–11635*	This work
GTS28	DSM 2542 Δ*11720*	This work
GTS29	DSM 2542 Δ*12555*	This work
GTS30	DSM 2542 Δ*15110*	This work
GTS31	DSM 2542 Δ*15920*	This work
GTS32	DSM 2542 Δ*18760–18770*	This work
*E. coli* DH5α^TM^	F- Φ80*lacZ*ΔM15 Δ(*lacZYA-argF*) U169 *recA1 endA1 hsdR17*(rk−, mk+) *phoA supE*44 *thi-1 gyrA*96 *relA1* λ−	ThermoFisher Scientific

*DSM* Deutsche sammlung von mikroorganismen (German Collection of Microorganisms), *Kan*^R^ kanamycin resistance.

### Knockout of individual PTS gene clusters

**Figure 4:**
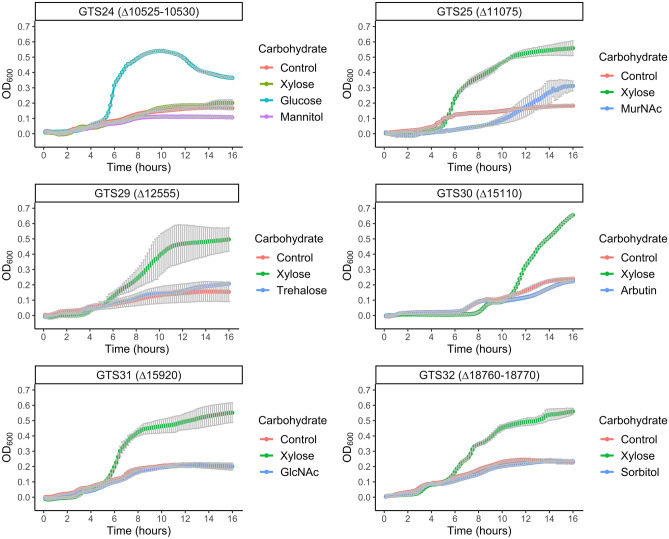
Growth curves of *P. thermoglucosidasius* DSM 2542 ∆*10525–10530,* ∆*11075,* ∆*12555,* ∆*15110,* ∆*15920,* and ∆*18760–18770* on TMMYE medium without (control) or with supplemented carbohydrates. Only strain:carbohydrate pairs with significantly impaired growth are displayed. The full set of growth curves can be found in the supplementary material. Data presented are mean values based on at least three replicates. Error bars indicate standard deviations.

To understand the importance of each individual PTS gene cluster in the carbohydrate metabolism of *P. thermoglucosidasius*, a minimum of one PTS element was knocked out of each cluster (Fig. 2). The resulting 15 deletion variants were subjected to the same growth assays as ∆*ptsI* and WT described above (see Supplementary Figs. 1–15). Cultures of the six knockout strains (AOT13_10525-10530; AOT13_11075; AO13_12555; AO13_15110; AO13_15920 and AO13_18760-18770) had no noticeable growth (or deficient growth in the case of AOT13_11075) when supplementing with a specific carbohydrate suggesting a direct relationship between the knocked-out PTS and the carbohydrate (Fig. 4). Strains ∆*10525-10530*, ∆*11075,* ∆*12555*, ∆*15110*, ∆*15920*, and ∆*18760-18770* exhibited significantly impaired growth when supplemented with mannitol, *N*-acetylmuramic acid, trehalose, arbutin, *N*-acetylglucosamine, and sorbitol, respectively. Strain ∆*10525-10530* showed inhibited growth on xylose as well as the mentioned mannitol, a phenotype that was not observed for the strain ∆*ptsI*. In addition, the growth of this mutant when supplemented with most of the carbohydrates showed to be lower compared with the WT strain (see Supplementary Fig. 7).

## Discussion

*P. thermoglucosidasius-*related species hold potential to produce valuable compounds from a broad variety of carbon sources. Rapid growth, availability of genetic tools, and, most importantly, its thermophilic nature, make this strain a prominent workhorse for large-scale industrial production. In light of the features, in this study, we systematically investigated the role of the PTSs of *P. thermoglucosidasius* in the transport of 15 common molecules used as carbon feedstock by knocking out both common and specific PTS components. The elimination of some components of the PTS systems affected carbon substrate utilization on the host strain. The extent of this impact is variable, and carbon substrate-dependent.

Knockout of the shared EI enzyme resulted in abolished growth for all tested carbohydrates except maltose, xylose, and *N*-acetylmuramic acid. Although many carbohydrates depend on PTS-mediated translocation with subsequent phosphorylation, other transporters are also known for transporting carbohydrates in bacteria. Here, the ATP-binding cassette (ABC) and the major facilitator superfamily (MFS) transporters also play a major role in the sugar uptake in prokaryotes^21–23^. The uptake of maltose has previously been described to occur through an ABC transporter in other bacterial species^24–27^. However, previous works on *Bacillus* have had contradictory results regarding maltose transport. Reizer *et al.*^27^ showed that inactivation of the putative EIIBC (*malP*) in *B. subtilis* resulted in a seven-fold increase of the doubling time in a minimal medium supplemented with maltose. Similarly, Schönert *et al.*^28^ have shown a lack of [^14^C]maltose uptake in cell suspensions of *B. subtilis* Δ*malP* after growth in LB supplemented with 1% maltose. Contrary, both in *B. subtilis* and in *B. licheniformis*, another major industrial Gram-positive bacteria, it has been suggested that maltose is transported by a proton symport mechanism, which does not occur via the PTS but is regulated by the PTS, and further metabolized through a maltose phosphorylase^29,30^. Furthermore, complementation of an *E. coli* strain deficient for maltose transport genes, with the MFS transporter MalA from *Geobacillus stearothermophilus* suppressed the growth defects on maltose^31^. A homologous genomic region encoding the same putative genes, including an MFS transporter homologue to MalA, is also found in *P. thermoglucosidasius* (AOT13_18465). In addition, a homologue of the maltose/maltodextrin transport system permease protein MalG from *E. coli*, part of the MalEFGK ABC transporter complex^32^, is also found in *P. thermoglucosidasius* (AOT13_07020). The results presented in our work, the presence of a MalA homologue, and the presence of a putative maltodextrin phosphorylase in the genome of *P. thermoglucosidasius* DSM 2542 (AOT13_18705), suggest a PTS-independent pathway.

Xylose is transported across the cell membrane either mediated by a PTS-independent mechanism or by a PTS-dependent but phosphorylation-independent mechanism. Facilitated diffusion of xylose catalyzed by the enzyme II complex (EII) of the PTS specific to mannose, has previously been reported in three species of lactobacilli (*L. pentosus, L. plantarum,* and *L. casei*)^33^. The transport was demonstrated to be independent of phosphorylation, which could explain why the growth of ∆*ptsI* supplemented with xylose remains unaffected. Genome analysis of six *Geobacillus* strains showed that xylose transport and metabolism are encoded in a gene cluster containing ABC transporters^34^. It has also been shown that *xyl* genes in thermophilic *Bacillus* sp. are clustered encoding *xylO* (ATP-binding protein), *xylP* (xylose permease), *xylA* (xylose isomerase), *xylB* (xylulose kinase)^35^, and although *P. thermoglucosidasius* DSM 2542 has an operon with both homologues to *xylA* and *xylB* (AOT13_11570 and AOT13_11575 respectively), it lacks homologues of *xylO* and *xylP*. Since strain *∆10525–10530* is not able to grow when supplemented with xylose, the EII complex encoded in that gene cluster probably catalyzes the facilitated diffusion of xylose with a similar mechanism as reported for lactobacilli, which would further be metabolized through the operon *xylAB*. In addition to the described mechanisms, xylose has been shown to be transported by AraE (part of the MFS) in *Bacillus subtilis*^23^*.* However, no homologue of AraE is found in *P. thermoglucosidasius* to our knowledge.

In both Gram-negative and -positive bacteria, *N*-acetylmuramic acid is mainly transported across the membrane by the PTS MurP and subsequently phosphorylated yielding the 6-phospho sugar utilized for peptidoglycan formation, or as carbon source through the *N*-acetylglucosamine-6P degradation pathway^36,37^. This seems to be the case also for *P. thermoglucosidasius* DSM 2542, which contains a putative operon encoding homologues to the regulator MurR, the etherase MurQ and the transporter MurP. Moreover, this is supported by the diminished growth of the strain *∆11075* on this carbon source.

Still remain to be determined which mechanism allows the partial metabolism of *N*-acetylmuramic acid observed on the strains *∆ptsI* and *∆11075*. In bacteria, the first PTS-independent *N*-acetylmuramic acid transporter has been identified in the periodontal pathogen *Tannerella forsythia*^38^. This organism contains an operon encoding a specific *N*-acetylmuramic acid transporter, a sugar kinase, and a MurQ etherase. No homologues of *T. forsythia* transporter and kinase are found in the genome of *P. thermoglucosidasius*, however, some of its many uncharacterized transporters and kinases could have unspecific activity towards *N*-acetylmuramic acid.

For all carbon sources tested, except for the three discussed above, growth was inhibited by the deletion of *ptsI*. While this is not generally surprising, we could have expected PTS-independent growth on glycerol. This triol is known to be transported both in Gram-negative and Gram-positive bacteria by energy-independent diffusion mediated by GlpF, a conserved glycerol uptake facilitator^39,40^. Although *P. thermoglucosidasius* DSM 2542 encodes the corresponding homologue (AOT13_09870), the mutant ∆*ptsI* had impaired growth when the medium was supplemented with glycerol. Previous studies also report inhibited growth on glycerol by mutants of Gram-positive and Gram-negative bacteria defective in one of the common enzymes of the PTS^41–46^. The mechanisms involved in this regulation are different in Gram-negative compared to Gram-positive bacteria but both are mediated by the glycerol kinase responsible for the formation of glycerol-3-phosphate trapping the substrate in the cell upon uptake^41^. For Gram-positive bacteria, the glycerol kinase was found to be phosphorylated by PEP and the common enzymes of the PTS; EI and HPr, causing an increase in glycerol kinase activity^41,43,46,47^. In fact, the His-232 of glycerol kinase from *Enterococcus casseliflavus* has been identified as the site of PEP-dependent PTS-catalyzed phosphorylation^47^. Given that glycerol kinase from *P. thermoglucosidasius* DSM 2542 (AOT13_09875) shares 64% homology to the enzyme from *E. casselliflavus*, and it contains the highly conserved histidine-232 residue, we suggest the same regulatory mechanism.

The findings of this study could have important implications for the future scalability and industrial applications of *P. thermoglucosidasius* as a cell factory or whole-cell biocatalysis. By identifying the specific PTS systems responsible for the transport and phosphorylation of various carbon substrates, this study lays the groundwork for future engineering efforts aimed at enhancing the strain's ability to efficiently utilize diverse carbon sources. Such efforts could potentially lead to the development of a highly versatile whole-cell biocatalyst capable of converting a wide range of substrates into valuable products. Overall, this study provides valuable insights into the metabolic capabilities of *P. thermoglucosidasius.*

## Materials and methods

### Bacterial strains and plasmids

The strains and plasmids used in this study are listed in Table 1 and Supplemental table 1 respectively. *P. thermoglucosidasius* DSM 2542 strains were routinely grown at 60 °C under agitation (200 rpm) on SPY medium (per litre: 16 g soy peptone, 10 g yeast extract, 5 g NaCl) and plated on Trypticase Soy Agar (TSA) plates (Becton Dickinson, US) unless stated differently. *E. coli* DH5α^TM^ subcloning efficiency^TM^ (ThermoFisher Scientific, Germany), was used as host in cloning experiments and grown in Luria–Bertani medium at 37 °C under agitation. *E. coli* DH5α^TM^ and *P. thermoglucosidasius* DSM 2542 transformants were selected with kanamycin (6.25 μg mL^−1^ and 12.5 μg mL^−1^ respectively, given the resulting promotor strength in each particular host).

### Bioinformatic analysis

To identify PTS-related genes in the genome of *P. thermoglucosidasius* DSM 2542, the hmmscan tool from the HMMer suite^48^ was used with the following profiles downloaded from the Pfam database^49^. PF00358.23; PF00359.25; PF00367.23; PF02255.19; PF02378.21; PF02896.21; PF03608.16; PF03611.17; PF03612.17; PF03829.16; PF05524.16; PF07663.14, using the trusted cutoffs provided within the individual profiles.

### DNA manipulation, oligonucleotides and sequencing

PCR primers (Supplemental table 2) were synthesized by IDT (USA). 11M2 and 12 backbone primers or custom primers hybridizing within the appropriate DNA fragments were used for checking plasmid assembly, integration site and verifying genome deletions. All PCR reactions were performed with the Phusion U Hot Start polymerase (ThermoFisher Scientific, Germany), and colony PCR reactions with OneTaq® (New England Biolabs, US). DNA sequencing was carried out by Eurofins Scientific (Luxembourg). Sequence analyses were carried out with SnapGene Viewer (Dotmatics) and sequence similarities were analyzed with BLAST^50^.

### Preparation of *P. thermoglucosidasius* DSM 2542 electrocompetent cells

To make electrocompetent cells, *P. thermoglucosidasius* DSM 2542 was initially inoculated into 50 mL of SPY and incubated at 60 °C with shaking (200 rpm) until an OD600 of ~1.5. The culture was then diluted to an OD600 of 0.5 in a new flask with 30 mL of fresh SPY and incubated at 60 °C with shaking (200 rpm) until an OD600 of ~1.7. After 10 minutes of incubation on ice, the culture was divided into two aliquots. The aliquots were washed consecutively four times in 15, 10, 10, and 5 mL of ice-cold electroporation buffer (0.5 M mannitol, 0.5 M sorbitol, 10% glycerol), and finally resuspended in 2 mL of electroporation buffer. The final cell suspensions were aliquoted (60 µl) in pre-chilled Eppendorf tubes and stored frozen at −80 °C.

### Construction of recombinant strains

Construction of the mutant strains was performed by two-step allelic exchange through homologous recombination, exploiting the native machinery of *P. thermoglucosidasius* DSM 2542^51^. DNA fragments containing flanking regions of the targeted genes designed to include only the start and stop codons of the knockout-target genes were obtained by PCR using *P. thermoglucosidasius* DSM 2542 chromosomal DNA and the corresponding oligonucleotide pairs. The PCR products were purified using a NucleoSpin Gel and PCR kit (Macherey-Nagel, Germany) and cloned into the backbone of pMTL61110 (obtained by PCR using primers 23 and 24d) by USER cloning (New England Biolabs, US). Chemically competents *E. coli* DH5α were transformed by heat shock with 3 μl of the USER reactions and after 1h recovery cells were plated on LB with kanamycin. The resulting plasmids were purified using a NucleoSpin Plasmid kit (Macherey-Nagel, Germany), and its sequence verified (Eurofin Genomics). *P. thermoglucosidasius* DSM 2542 electrocompetents^6^ were transformed with each of these plasmids using a single electric pulse in a Bio-Rad GenePulser Xcell (10 μF, 600 Ω, 25 kV/cm) and recovered in 1mL of pre-warmed SPY supplemented with 1% glycerol at 52 °C with agitation (200 rpm) for 3 hours. Selection of kanamycin-resistant colonies was done on TSA plates with 12.5 μg ml^−1^ kanamycin overnight at 52 °C. A kanamycin-resistant colony from each transformation was incubated overnight at 62 °C on SPY supplemented with kanamycin to force the first recombination and later plated on TSA plates with 12.5 μg ml^−1^ kanamycin. After confirming the right integration site by colonyPCR, the selected colonies were grown 3 days in 5mL of SPY without kanamycin at 60 °C with agitation (200 rpm), doing subcultures in fresh media each morning and evening. Cells were plated on TSA and incubated at 60 °C overnight. The next day the plates were replicaplated on TSA plus kanamycin. Antibiotic-sensitive clones were isolated and, among them, one for each gene was selected (GTS17-GTS32 strains) in which a second recombination event led to the excision of the plasmid and deletion of the targeted gene. DNA sequencing reactions of the appropriate PCR products (see Supplementary Fig. 16) were performed by Eurofins Genomics.

### Culture of *P. thermoglucosidasius* strains with 15 carbon substrates

Precultures of *P. thermoglucosidasius* strains were grown overnight at 60 °C under agitation (200 rpm) on sugar-free Thermophile Minimal Medium (TMM) supplemented with 3 g/L yeast extract (TMMYE). TMM is adapted from Fong et al.^52^ and contained the following sterile solutions, per litre: 930 mL Six Salts Solution (SSS), 40 mL of 1 M MOPS solution (pH = 8.2), 10 mL of 1 mM FeSO_4_ in 0.4 M tricine, 10 mL of 0.132 M K_2_HPO_4_, 10 mL of 0.953 M NH_4_Cl, 0.5 mL of 1 M CaCl_2_, 1x trace elements solution, and 1x Wolfe’s vitamin solution, with the final pH adjusted to 6.8. SSS contained, per litre: 4.95 g NaCl, 1.45 g Na_2_SO_4_, 0.25 g KCl, 0.04 g KBr, 1.85 g MgCl_2_·6H_2_O, and 0.89 g NaNO_3_. The trace element solution contains 1 g FeCl_3_·6H_2_O, 0.18 g ZnSO_4_·7H_2_O, 0.12 g CuCl_2_·2H_2_O, 0.12 g MnSO_4_·H_2_O, and 0.18 g CoCl_2_·6H_2_O. Finally, Wolfe’s vitamin solution contained, per litre: 10 mg Pyridoxine HCl, 5 mg Thiamine HCl, 5 mg Riboflavin, 5 mg Nicotinic acid, 5 mg Ca-D-(+)pantothenate, 5 mg p-Aminobenzoic acid, 5 mg Thiotic acid (Dithiolane Pentanoic acid), 2 mg Biotin, 2 mg Folic acid, and 0.1 mg Vitamin B12. Overnight cultures were diluted 1:12,5 in 200 μL of TMMYE with or without the following carbon substrates (80mM glycerol; 48mM xylose; 40 mM glucose, fructose, mannose, mannitol or sorbitol; 30mM *N*-acetyl-glucosamine; 26mM* N*-acetyl-muramic acid; 20mM cellobiose, maltose, sucrose, or trehalose; 8mM arbutin or salicin). All carbohydrates/glucosides were purchased from Sigma-Aldrich (USA) or Carbosynth (Compton, Berkshire, UK). Cultures were incubated in 96-well plates sealed with Titer-Tops® (Sigma-Aldrich, USA) at 60 °C with agitation (567 cpm) in an Epoch2 microplate spectrophotometer (BioTek, Agilent, USA), and bacterial growth was monitored every 10 minutes for 24 h measuring OD_600 nm_. At least three independent biological replicates for each growth curve were obtained. Results were expressed as means ± standard deviations.

### Figures and data analysis

Figure 1 was created with BioRender (https://www.BioRender.com). Data analysis and illustrations were prepared in R (https://www.R-project.org/) using RStudio (https://www.RStudio.com).

## Supplementary Information

Supplementary Information.

## Data Availability

All data generated or analyzed during this study are included in this published article (and its Supplementary Information files).
