# Supplementary material for: Functional characterization of the phosphotransferase system in Parageobacillus thermoglucosidasius
Source: Sci Rep. 2023 May 2;13:7131. doi: 10.1038/s41598-023-33918-1 (PMC10154347; doi:10.1038/s41598-023-33918-1)
Supplement: Supplementary file 1 — Supplementary Information. [file 41598_2023_33918_MOESM1_ESM.pdf]

Functional Characterization of the Phosphotransferase System in Parageobacillus thermoglucosidasius

Gonzalo N. Bidart[1], Hani Gharabli[1], Ditte Hededam Welner[1,*]

Supplementary material

Supplementary Figure S1 – Strain GTS18 (Δ03265) 2

Supplementary Figure S2 – Strain GTS19 (Δ03410) 2

Supplementary Figure S3 – Strain GTS20 (Δ04155) 2

Supplementary Figure S4 – Strain GTS21 (Δ05250) 3

Supplementary Figure S5 – Strain GTS22 (Δ08095) 3

Supplementary Figure S6 – Strain GTS23 (Δ10220-10230) 3

Supplementary Figure S7 – Strain GTS24 (Δ10525-10530) 4

Supplementary Figure S8 – Strain GTS25 (Δ11075) 4

Supplementary Figure S9 – Strain GTS26 (Δ11155) 4

Supplementary Figure S10 – Strain GTS27 (Δ11625-11635) 5

Supplementary Figure S11 – Strain GTS28 (Δ11720) 5

Supplementary Figure S12 – Strain GTS29 (Δ12555) 5

Supplementary Figure S13 – Strain GTS31 (Δ15110) 6

Supplementary Figure S14 – Strain GTS32 (Δ15920) 6

Supplementary Figure S15 – Strain GTS33 (Δ18760-18770) 6

Supplementary Figure S16 8

Supplementary Table 1 – Plasmids used in this study 9

Supplementary Table 2 – Primers used in this study 10

Supplementary Figure S1 – Strain GTS18 (Δ03265)

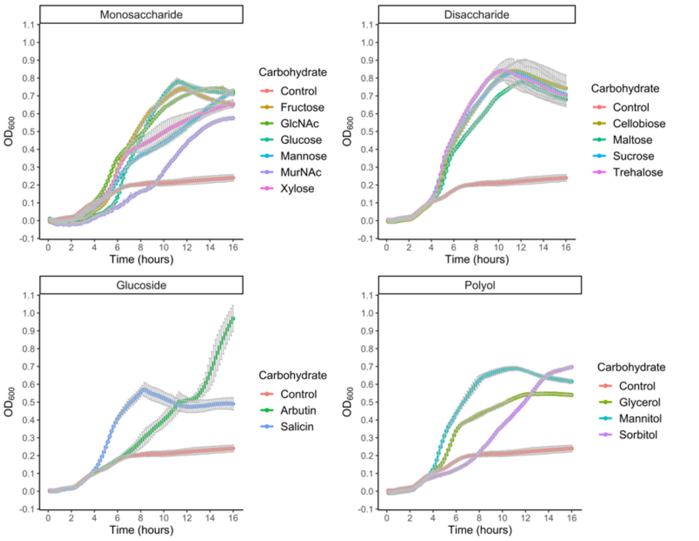

Supplementary Figure S2 – Strain GTS19 (Δ03410)

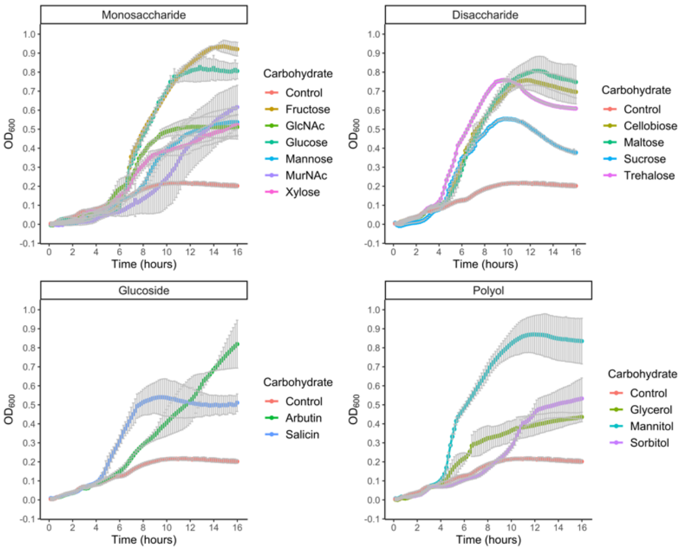

Supplementary Figure S3 – Strain GTS20 (Δ04155)

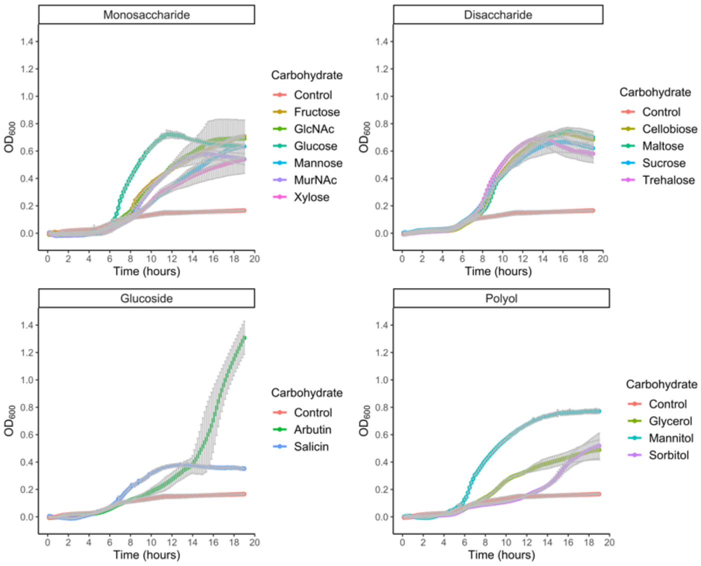

Supplementary Figure S4 – Strain GTS21 (Δ05250)

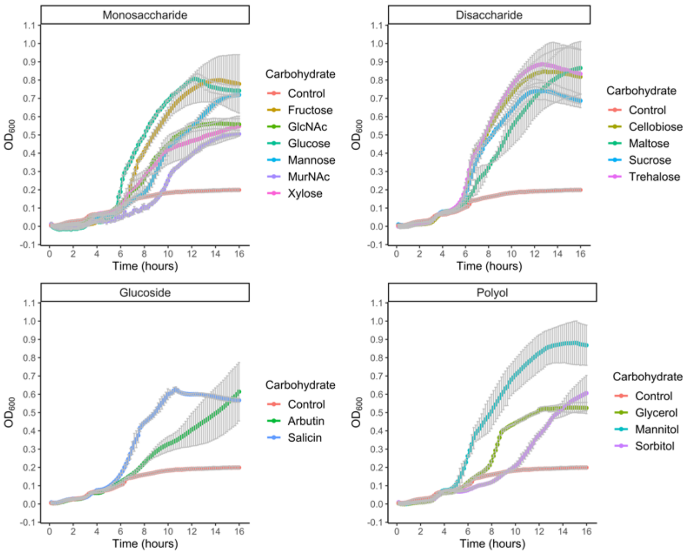

Supplementary Figure S5 – Strain GTS22 (Δ08095)

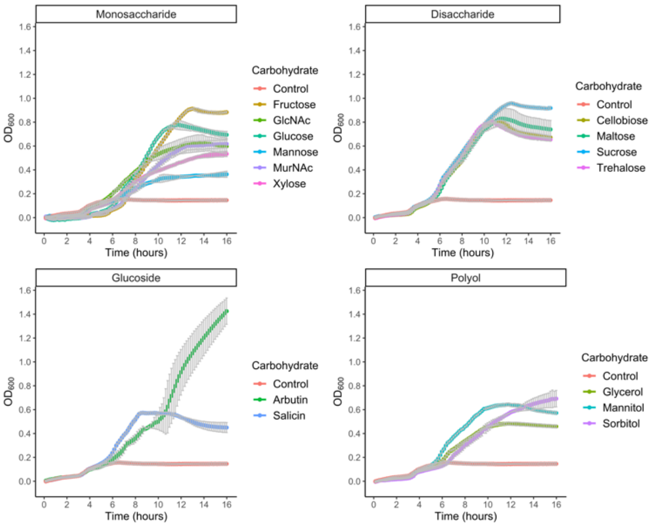

Supplementary Figure S6 – Strain GTS23 (Δ10220-10230)

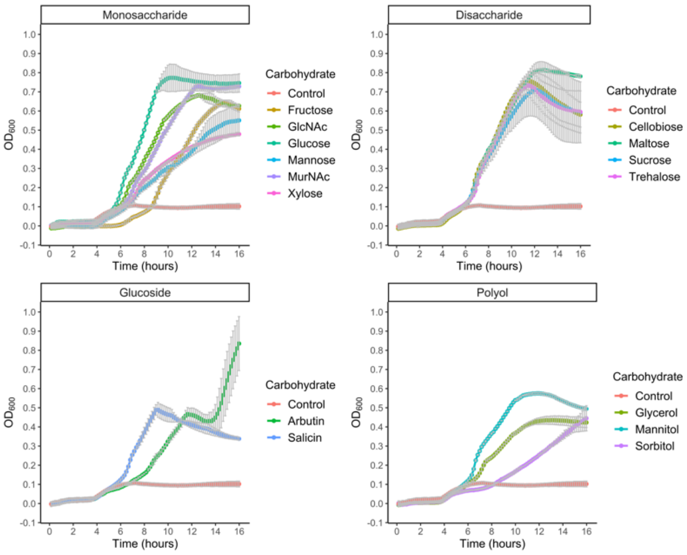

Supplementary Figure S7 – Strain GTS24 (Δ10525-10530)

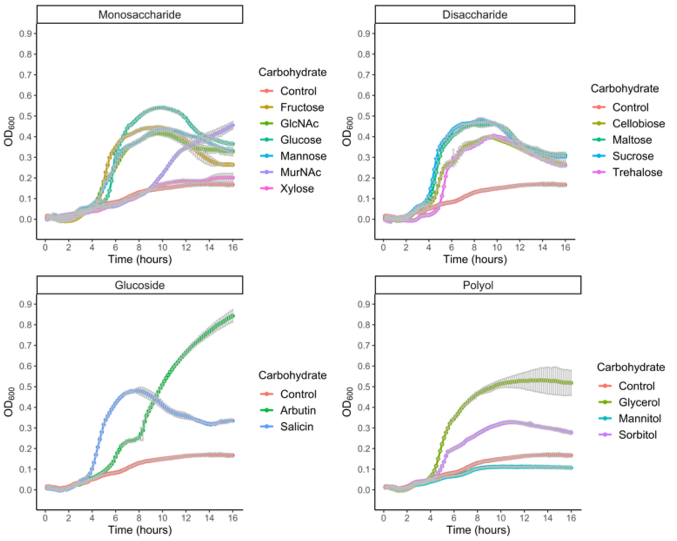

Supplementary Figure S8 – Strain GTS25 (Δ11075)

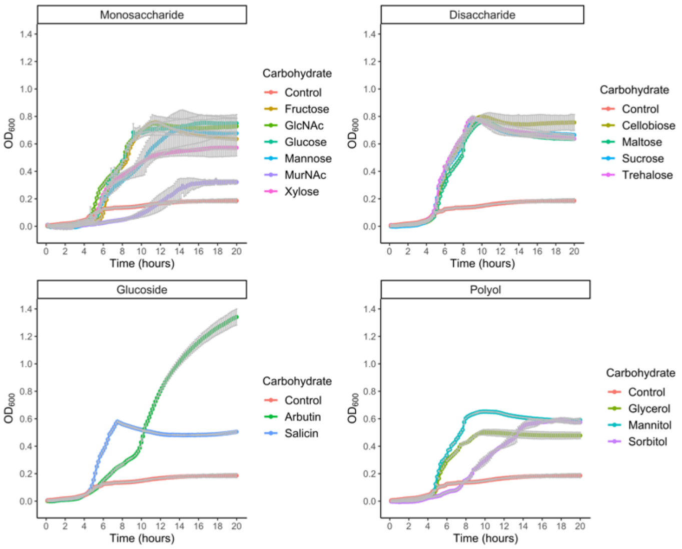

Supplementary Figure S9 – Strain GTS26 (Δ11155)

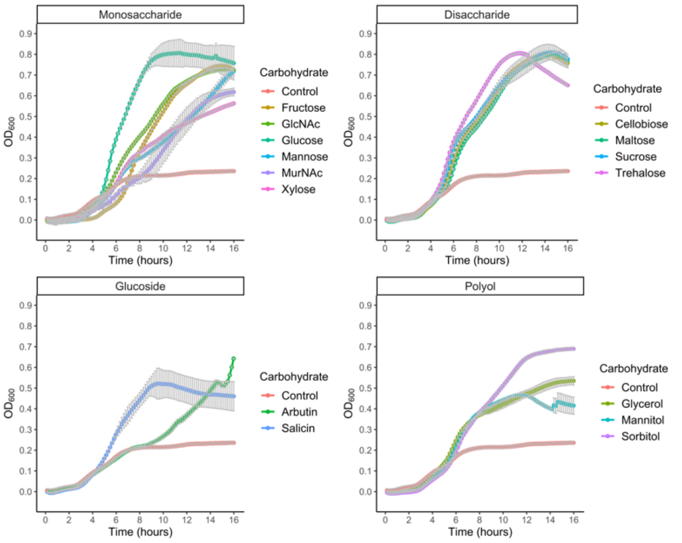

Supplementary Figure S10 – Strain GTS27 (Δ11625-11635)

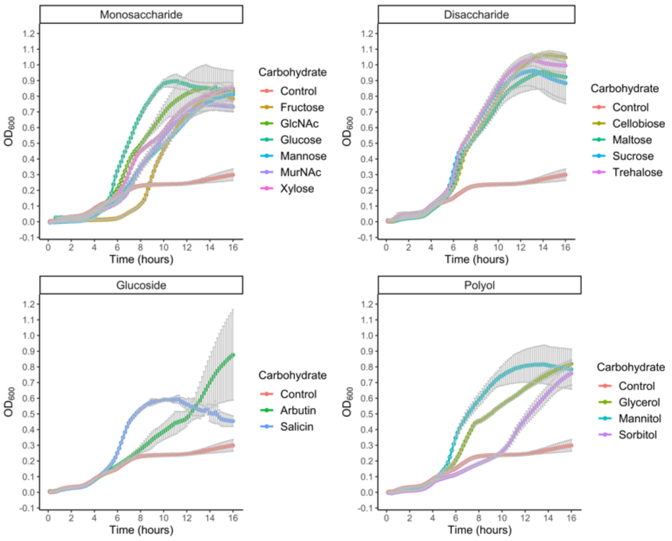

Supplementary Figure S11 – Strain GTS28 (Δ11720)

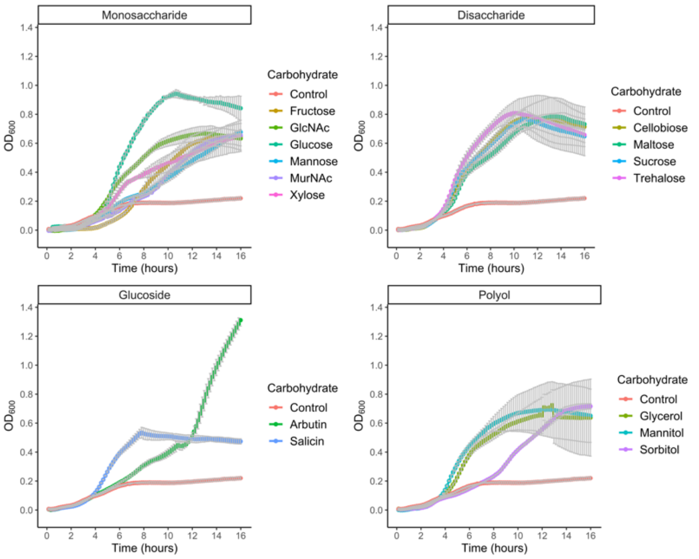

Supplementary Figure S12 – Strain GTS29 (Δ12555)

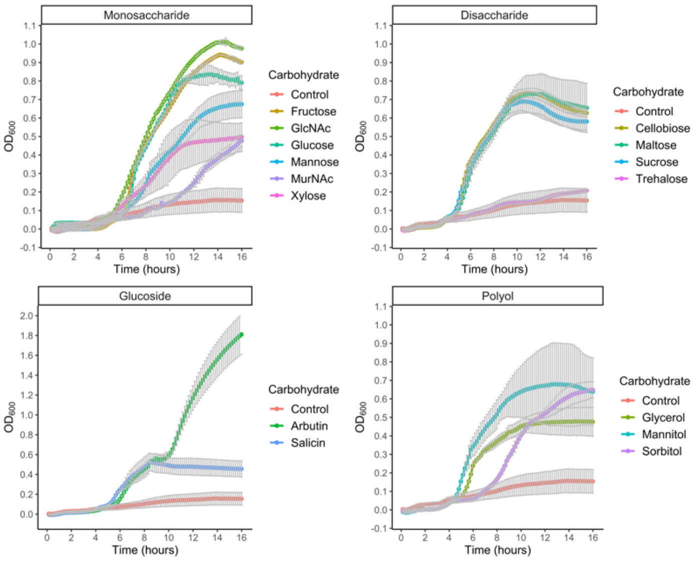

Supplementary Figure S13 – Strain GTS31 (Δ15110)

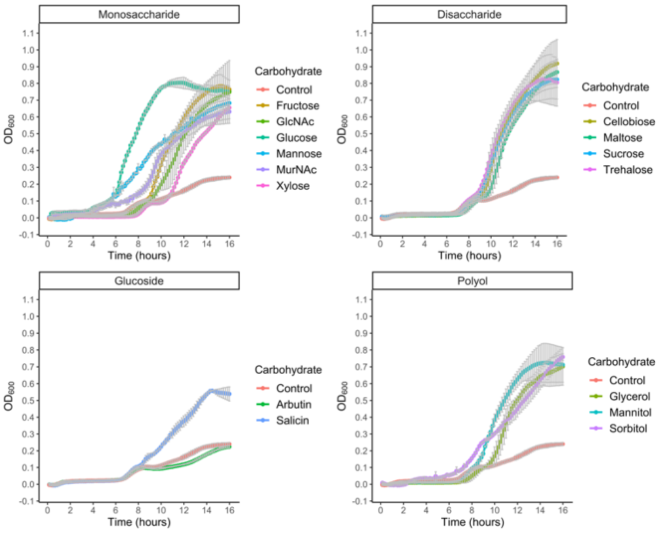

Supplementary Figure S14 – Strain GTS32 (Δ15920)

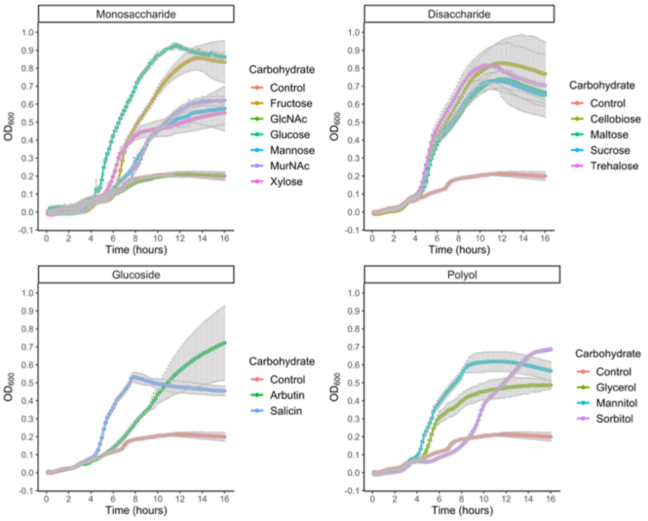

Supplementary Figure S15 – Strain GTS33 (Δ18760-18770)

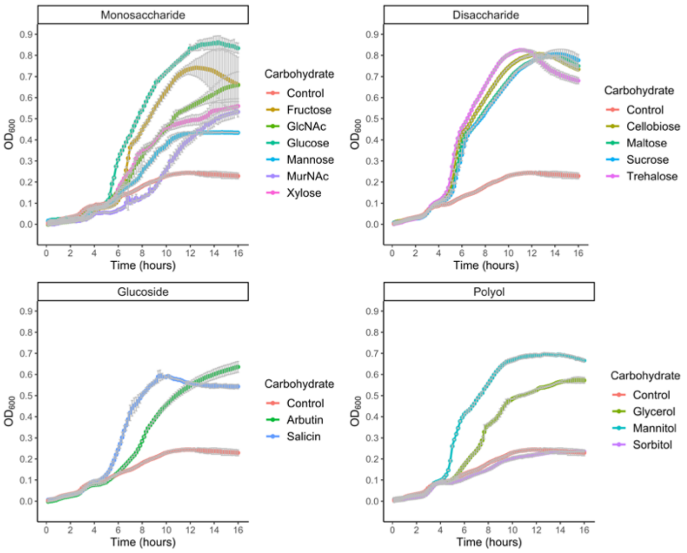

Legend for supplementary Figures S1 to S15:

Growth curves of mutant strains of *P. thermoglucosidasius* on TMMYE medium without (control) or with supplemented carbohydrates (Fructose, Glc*N*Ac, Glucose, Mannose, Mur*N*Ac, Xylose; Cellobiose, Maltose, Sucrose, Trehalose; Arbutin, Salicin; Glycerol, Mannitol or Sorbitol).

Supplementary Figure S16

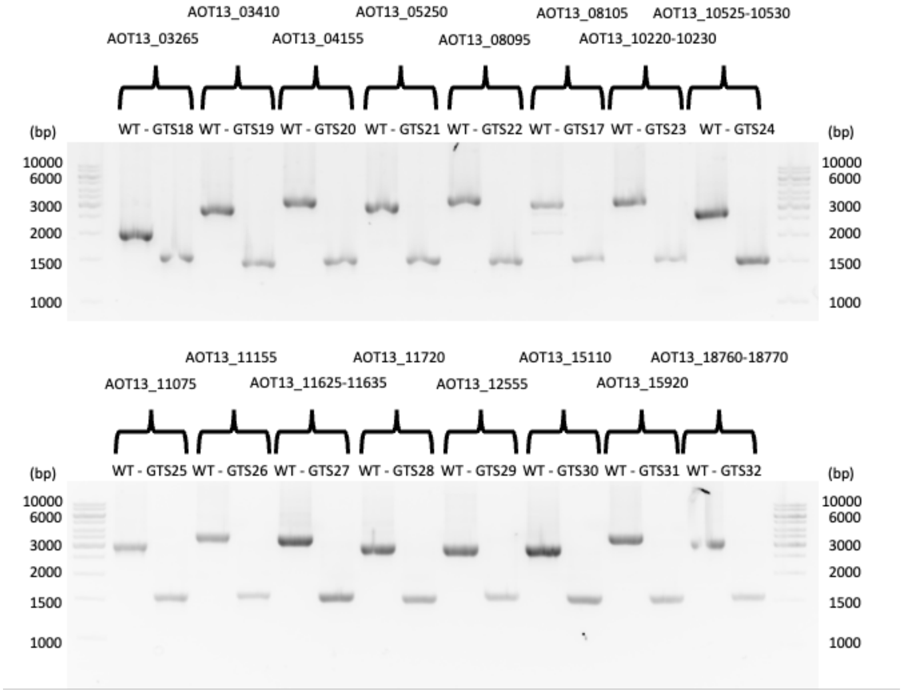

Supplementary Figure S16. Colony PCRs of the targeted loci in the mutant strains, prior sequencing verification. Parental strain DSM2542 (WT) used as control.

Supplementary Table 1 – Plasmids used in this study

Plasmid	Relevant genotype or properties	Source
pMTL61110	Integrative shuttle vector carrying Kan^R for *P. thermoglucosidasius*	Sheng *et al.* 2017[51]
pGeo-Δ*ptsI*	pMTL61110- carrying flanking regions of *ptsI*	This work
pGeo-Δ*03265*	pMTL61110- carrying flanking regions of AOT13_03265	This work
pGeo-Δ*03410*	pMTL61110- carrying flanking regions of AOT13_03410	This work
pGeo-Δ*04155*	pMTL61110- carrying flanking regions of AOT13_04155	This work
pGeo-Δ*05250*	pMTL61110- carrying flanking regions of AOT13_05250	This work
pGeo-Δ*08095*	pMTL61110- carrying flanking regions of AOT13_08095	This work
pGeo-Δ*10220-10230*	pMTL61110- carrying flanking regions of AOT13_10220 to AOT13_10230	This work
pGeo-Δ*10525-10530*	pMTL61110- carrying flanking regions of AOT13_10525 to AOT13_10530	This work
pGeo-Δ*11075*	pMTL61110- carrying flanking regions of AOT13_11075	This work
pGeo-Δ*11155*	pMTL61110- carrying flanking regions of AOT13_11155	This work
pGeo-Δ*11625-11635*	pMTL61110- carrying flanking regions of AOT13_11625 to AOT13_11635	This work
pGeo-Δ*11720*	pMTL61110- carrying flanking regions of AOT13_11720	This work
pGeo-Δ*12555*	pMTL61110- carrying flanking regions of AOT13_12555	This work
pGeo-Δ*15110*	pMTL61110- carrying flanking regions of AOT13_15110	This work
pGeo-Δ*15920*	pMTL61110- carrying flanking regions of AOT13_15920	This work
pGeo-Δ*18760-18770*	pMTL61110- carrying flanking regions of AOT13_18760 to AOT13_18770	This work

DSM, Deutsche Sammlung von Mikroorganismen (German Collection of Microorganisms); Kan^R, kanamycin resistance.

Supplementary Table 2 – Primers used in this study

No.	Sequence 5'-3'	Description
23	ACCCGGGGUTCCTCTAG	Forward primer to amplify backbone fragment from pMTL61110
24d	AATTCGUAATCATGGTCATATGGATACAGCG	Reverse primer to amplify backbone fragment from pMTL61110
11M2	GGCCGCTGTATCCATATGACCATG	Forward primer for sequencing of homologous arms region in plasmid, and to check integration sites
12	GTTGTAAAACGACGGCCAGTGC	Reverse primer for sequencing of homologous arms region in plasmid, and to check integration sites
AOT13_ptsI-LF-Fw	ACGAATUCAAAAAATGATGGGGGACGGCTTTG	Forward primer to amplify *ptsI* left homologous arm from gDNA
AOT13_ptsI-LF-Rv	ACCGCTAAUTCCATTATTGTGCAAGACCTTCCTTATTTAATGTTTCTGTC	Reverse primer to amplify *ptsI* left homologous arm from gDNA
ptsIRF-Fw	ATTAGCGGUGGAGTCATATTCCACCG	Forward primer to amplify *ptsI* right homologous arm from gDNA
ptsI-RF-Rv	ATCCCCGGGUATCAACAAAGCCATCAATGCGATAAGTTTTG	Reverse primer to amplify *ptsI* right homologous arm from gDNA
SeqptsIF	AGAATCTGGAATTCACGCTCGC	Forward primer for sequencing of *ptsI* genomic region
SeqptsIR	TGGTCAAGTTCCGACCATGCTG	Reverse primer for sequencing of *ptsI* genomic region
AOT13_03265-LF-Fw	ACGAATUAGTCATTGCAGTCGTTTGCCTCG	Forward primer to amplify AOT13_03265 left homologous arm from gDNA
AOT13_03265-LF-Rv	ACATGTUGTTCCCTCCCGTTTTGTA	Reverse primer to amplify AOT13_03265 left homologous arm from gDNA
03265-RF-Fw	AACATGUAAACAGAAATATTTTTGTATAATGAAATTATGGTGAAC	Forward primer to amplify *spo0A* right homologous arm from gDNA
03265-RF-Rv	ATCCCCGGGUATATCCGCGTCGCGATGCG	Reverse primer to amplify AOT13_03265 right homologous arm from gDNA
Seq03265F	GGAGGGGAAAACCACAAAACGG	Forward primer for sequencing of AOT13_03265 genomic region
Seq03265R	GCGTCATCCACTTTAGCGACG	Reverse primer for sequencing of AOT13_03265 genomic region
AOT13_03410-LF-Fw	ACGAATUACAATCATATTCATATTGCTTTAACCGATCACTTATCATTTG	Forward primer to amplify AOT13_03410 left homologous arm from gDNA
AOT13_03410-LF-Rv	ATGACUGAATTGTGAACCCCTCTCTTTTATTTTTTAAAAAAGCAAAAAAG	Reverse primer to amplify AOT13_03410 left homologous arm from gDNA
03410-RF-Fw	AGTCAUGCCTGATTCGCTGCG	Forward primer to amplify AOT13_03410 right homologous arm from gDNA
03410-RF-Rv	ATCCCCGGGUCAAACAAGGAGCACTTCTTGTCCGTC	Reverse primer to amplify AOT13_03410 right homologous arm from gDNA
Seq03410F	TACTGCAAACGCAGGCAAGAC	Forward primer for sequencing of AOT13_03410 genomic region
Seq03410R	TGAGAACGACAGTATCGCGCAC	Reverse primer for sequencing of AOT13_03410 genomic region
AOT13_04155-LF-Fw	ACGAATUAGATAAAGACGATGAAGTCTTTTGGCTG	Forward primer to amplify AOT13_04155 left homologous arm from gDNA
AOT13_04155-LF-Rv	ATTACAUCGTTTTTAGTTCCACGGTAATATC	Reverse primer to amplify AOT13_04155 left homologous arm

		from gDNA
04155-RF-Fw	ATGTAAUTATAGACAAAAAAATATGGTTATGGGTTCATG	Forward primer to amplify AOT13_04155 right homologous arm from gDNA
04155-RF-Rv	ATCCCCGGGUTAGAAGATGATCGCAATGCCTATTCATTTC	Reverse primer to amplify AOT13_04155 right homologous arm from gDNA
Seq04155F	CGGATTTTTTCTGCAGCTTGTATGATCG	Forward primer for sequencing of AOT13_04155 genomic region
Seq04155R	CGGACAAGCACTTGGTCACC	Reverse primer for sequencing of AOT13_04155 genomic region
AOT13_05250 -LF-Fw	ACGAATUCGCTCCTAAAACAGAGGAAGTGAAC	Forward primer to amplify AOT13_05250 left homologous arm from gDNA
AOT13_05250 -LF-Rv	ACATGTUCACTCACTCCCTTTCTTAAATTTT	Reverse primer to amplify AOT13_05250 left homologous arm from gDNA
05250-RF-Fw	AACATGUAACGATCATGATGAATAAGAAGTTGGG	Forward primer to amplify AOT13_05250 right homologous arm from gDNA
05250-RF-Rv	ATCCCCGGGUTAATATCTCACCGTCATTTCTTTGCGG	Reverse primer to amplify AOT13_05250 right homologous arm from gDNA
Seq05250F	CGCCAACAACCAATCCGC	Forward primer for sequencing of AOT13_05250 genomic region
Seq05250R	CGCGGATATCTGTCCACCATCC	Reverse primer for sequencing of AOT13_05250 genomic region
AOT13_08095 -LF-Fw	ACGAATUAGCTTATATCCGTTGGAATATGAAATTGCG	Forward primer to amplify AOT13_08095 left homologous arm from gDNA
AOT13_08095 -LF-Rv	ATTACATUTTTATAACCTCCTTCTCTCTATAAG	Reverse primer to amplify AOT13_08095 left homologous arm from gDNA
08095-RF-Fw	AATGTAAUAAAGTGAATTTTTCTTGTAAATTTACTTTTGTC	Forward primer to amplify AOT13_08095 right homologous arm from gDNA
08095-RF-Rv	ATCCCCGGGUTCTTTCATATACTCATTATCCATCGCTTCG	Reverse primer to amplify AOT13_08095 right homologous arm from gDNA
Seq08095F	GCTTGATTTAAAAAACCCGTTCTTGGTCG	Forward primer for sequencing of AOT13_08095 genomic region
Seq08095R	ACCCGCTTTGTCACATCGC	Reverse primer for sequencing of AOT13_08095 genomic region
AOT13_10220 -10230-LF-Fw	ACGAATUATTTGCACCGGTGTTTTTAGAAACACC	Forward primer to amplify AOT13_10220-10230 left homologous arm from gDNA
AOT13_10220 -10230-LF-Rv	ATTACACUTATTTATCACCCCAATATTTCTAAAC	Reverse primer to amplify AOT13_10220-10230 left homologous arm from gDNA
10220-10230-RF-Fw	AGTGTAAUAATAGATTTTTAGACTCCTTCTGTTTC	Forward primer to amplify AOT13_10220-10230 right homologous arm from gDNA
10220-10230-RF-Rv	ATCCCCGGGUTCCATCTGTTAAACTTTTTACAATATGGTTGATG	Reverse primer to amplify AOT13_10220-10230 right homologous arm from gDNA
Seq10220-10230F	TAGACTCATTATCAATCAGGGAATTCCAAG	Forward primer for sequencing of AOT13_10220-10230 genomic region
Seq10220-10230R	TCCTGCGCATTCAAAGACAAC	Reverse primer for sequencing of AOT13_10220-10230 genomic region
AOT13_10525 -10530-LF-Fw	ACGAATUGGAACATGTTGATTTTGTTGTTACAACC	Forward primer to amplify AOT13_10525-10530 left homologous arm from gDNA

AOT13_10525-10530-LF-Rv	ACATTTUTTGAACCTCCTTGTTTTTGCTC	Reverse primer to amplify AOT13_10525-10530 left homologous arm from gDNA
10525-10530-RF-Fw	AAAATGUAAGCACATAGGAACTCCCCCG	Forward primer to amplify AOT13_10525-10530 right homologous arm from gDNA
10525-10530-RF-Rv	ATCCCCGGGUAAGGCTTTTGCTGGCGCTTCC	Reverse primer to amplify AOT13_10525-10530 right homologous arm from gDNA
Seq10525-10530F	TGTCATTGACATCATTTCGGC	Forward primer for sequencing of AOT13_10525-10530 genomic region
Seq10525-10530R	GAGTGGAGGTTCGTCATGG	Reverse primer for sequencing of AOT13_10525-10530 genomic region
AOT13_11075-LF-Fw	ACGAATUTTGCATCTTTCCGGCAGGGG	Forward primer to amplify AOT13_11075 left homologous arm from gDNA
AOT13_11075-LF-Rv	ACTACATUTCAACCTCTCCCTTTCCTCTTTTA	Reverse primer to amplify AOT13_11075 left homologous arm from gDNA
11075-RF-Fw	AATGTAGUTGTAGGAGGCGTTTATGTTTTAATC	Forward primer to amplify AOT13_11075 right homologous arm from gDNA
11075-RF-Rv	ATCCCCGGGUCCGATAACGAAGCGGAATGACG	Reverse primer to amplify AOT13_11075 right homologous arm from gDNA
Seq11075F	CCGTTTCGAAAGAATTAACCCAAATCG	Forward primer for sequencing of AOT13_11075 genomic region
Seq11075R	GGAAAGCAGCTCATGTTGTCGG	Reverse primer for sequencing of AOT13_11075 genomic region
AOT13_11155-LF-Fw	ACGAATUGGGAATGAGAATTTCAAGATCCATTGGC	Forward primer to amplify AOT13_11155 left homologous arm from gDNA
AOT13_11155-LF-Rv	ACATATUGCACTTCCTCCTTTTCTTAAG	Reverse primer to amplify AOT13_11155 left homologous arm from gDNA
11155-RF-Fw	AATATGUAAGCGTTTGCTTACCTTTTTTAACATG	Forward primer to amplify AOT13_11155 right homologous arm from gDNA
11155-RF-Rv	ATCCCCGGGUGGTTCCGTTGCTTCCAGC	Reverse primer to amplify AOT13_11155 right homologous arm from gDNA
Seq11155F	GATCGACAATGGAGAAAAACGTTTCG	Forward primer for sequencing of AOT13_11155 genomic region
Seq11155R	GCCCGAGAAGTTTTTCTGTAACCG	Reverse primer for sequencing of AOT13_11155 genomic region
AOT13_11625-11635-LF-Fw	ACGAATUTCGGGAAACAAACCGGTTTGCAG	Forward primer to amplify AOT13_11625-11635 left homologous arm from gDNA
AOT13_11625-11635-LF-Rv	ACATTAUGATGCAACCTCCTTTATGTTC	Reverse primer to amplify AOT13_11625-11635 left homologous arm from gDNA
11625-11635-RF-Fw	ATAATGUAAAACGAGGTGGAAATCATGAAGC	Forward primer to amplify AOT13_11625-11635 right homologous arm from gDNA
11625-11635-RF-Rv	ATCCCCGGGUATCAGCAGGATGGCTGCTCC	Reverse primer to amplify AOT13_11625-11635 right homologous arm from gDNA
Seq11625-11635F	ATATTCATGCCGCAGTTCGCG	Forward primer for sequencing of AOT13_11625-11635 genomic region
Seq11625-11635R	CCGGATCTAAGAAGCTGCGG	Reverse primer for sequencing of AOT13_11625-11635 genomic region
AOT13_11720	ACGAATUTTTACGAGCAAATCATTGAAGAAGC	Forward primer to amplify

-LF-Fw		AOT13_11720 left homologous arm from gDNA
AOT13_11720 -LF-Rv	ATTACAUCAAAATACCCCCTTTTTTCATATGTTG	Reverse primer to amplify AOT13_11720 left homologous arm from gDNA
11720-RF-Fw	ATGTAAUAGAACATGCTGAGCGTGAATTG	Forward primer to amplify AOT13_11720 right homologous arm from gDNA
11720-RF-Rv	ATCCCCGGGUATAGACCGGTATAACACCATCGTC	Reverse primer to amplify AOT13_11720 right homologous arm from gDNA
Seq11720F	CGGCTCGGTGTAGAGAGC	Forward primer for sequencing of AOT13_11720 genomic region
Seq11720R	GCAAGCGCCATCATGACAAC	Reverse primer for sequencing of AOT13_11720 genomic region
AOT13_12555 -LF-Fw	ACGAATUCATGAATTTCTTCGGTTTCTTCCTGC	Forward primer to amplify AOT13_12555 left homologous arm from gDNA
AOT13_12555 -LF-Rv	ATTACAUCACGAAAACCCCCTAACTCCTT	Reverse primer to amplify AOT13_12555 left homologous arm from gDNA
12555-RF-Fw	ATGTAAUATATAAAACTATGTTCTAAATAATCATTAAACATGC	Forward primer to amplify AOT13_12555 right homologous arm from gDNA
12555-RF-Rv	ATCCCCGGGUGAAAACGTTGATCTTTAGACAACAAGTTGAC	Reverse primer to amplify AOT13_12555 right homologous arm from gDNA
Seq12555F	TGCAAAAACGTGACGACATTGTCG	Forward primer for sequencing of AOT13_12555 genomic region
Seq12555R	CCGGCGGCATCGAAC	Reverse primer for sequencing of AOT13_12555 genomic region
AOT13_15110 -LF-Fw	ACGAATUGAATCGAAGAGGGGGATAATTTAAAAAG	Forward primer to amplify AOT13_15110 left homologous arm from gDNA
AOT13_15110 -LF-Rv	ATCACAUAAGAATTCCTCCTTTTCTGTTAATT	Reverse primer to amplify AOT13_15110 left homologous arm from gDNA
15110-RF-Fw	ATGTGAUCCAGAACATAGAATTAAGATGTGTAG	Forward primer to amplify AOT13_15110 right homologous arm from gDNA
15110-RF-Rv	ATCCCCGGGUGAAAATTTCACAAAGAAATCAATACACTTCCG	Reverse primer to amplify AOT13_15110 right homologous arm from gDNA
Seq15110F	GATTAAATAAATGGTTCTTCGTTATTTACAATTAAATGAC	Forward primer for sequencing of AOT13_15110 genomic region
Seq15110R	CATCCAGTATTTCACTTTATCTTTATAACGTTTC	Reverse primer for sequencing of AOT13_15110 genomic region
AOT13_15920 -LF-Fw	ACGAATUGATTAACAAGCATTCCCCGCTTCC	Forward primer to amplify AOT13_15920 left homologous arm from gDNA
AOT13_15920 -LF-Rv	AAAGAAAUTCCTCCTCTTCATTATTTTCC	Reverse primer to amplify AOT13_15920 left homologous arm from gDNA
15920-RF-Fw	ATTTCTTUATGTAGCCAAAAAGAAAAGCGTTCCAGC	Forward primer to amplify AOT13_15920 right homologous arm from gDNA
15920-RF-Rv	ATCCCCGGGUGTTACTCCCGGCACAGTCC	Reverse primer to amplify AOT13_15920 right homologous arm from gDNA
Seq15920F	GGAGGCGTTATCGCTTGTGC	Forward primer for sequencing of AOT13_15920 genomic region
Seq15920R	ATGTTCATGGGCAATCCGAACC	Reverse primer for sequencing of

		AOT13_15920 genomic region
AOT13_18760 -18770-LF-Fw	ACGAATUTATATTGCACTTCCTCACACGC	Forward primer to amplify AOT13_18760-18770 left homologous arm from gDNA
AOT13_18760 -18770-LF-Rv	ATCACATUTTATTCACCCCAGAATTATTTT	Reverse primer to amplify AOT13_18760-18770 left homologous arm from gDNA
18760-18770-RF-Fw	AATGTGAUAAAAGTTTGTGGAAAGTGAATATATTGATG	Forward primer to amplify AOT13_18760-18770 right homologous arm from gDNA
18760-18770-RF-Rv	ATCCCCGGGUTGAATGACAGGCAAATGGCCGAAATAAATG	Reverse primer to amplify AOT13_18760-18770 right homologous arm from gDNA
Seq18760-18770F	AGCGCCTTAAAGAACGGGAAG	Forward primer for sequencing of AOT13_18760-18770 genomic region
Seq18760-18770R	CCACCCGCAAAATATGGAAGC	Reverse primer for sequencing of AOT13_18760-18770 genomic region
